# Connectomic analysis of unilateral dual-lead thalamic deep brain stimulation for treatment of multiple sclerosis tremor

**DOI:** 10.1093/braincomms/fcac063

**Published:** 2022-03-15

**Authors:** Joshua K. Wong, Bhavana Patel, Erik H. Middlebrooks, Justin D. Hilliard, Kelly D. Foote, Michael S. Okun, Leonardo Almeida

**Affiliations:** 1 Fixel Institute for Neurological Diseases, Department of Neurology, University of Florida, Gainesville, FL 32608, USA; 2 Department of Radiology, Mayo Clinic, Jacksonville, FL 32224, USA; 3 Fixel Institute for Neurological Diseases, Department of Neurosurgery, University of Florida, Gainesville, FL 32608, USA

**Keywords:** deep brain stimulation, multiple sclerosis, tremor, connectomics, dual lead

## Abstract

Tremor is a common symptom in multiple sclerosis and can present as a severe postural and action tremor, leading to significant disability. Owing to the diffuse and progressive nature of the disease, it has been challenging to characterize the pathophysiology underlying multiple sclerosis tremor. Deep brain stimulation of the ventralis intermedius and the ventralis oralis posterior thalamic nuclei has been used to treat medically refractory multiple sclerosis tremors with variable results. The aim of this study was to characterize multiple sclerosis tremor at the network level by applying modern connectomic techniques to data from a previously completed single-centre, randomized, single-blind prospective trial of 12 subjects who were treated with unilateral dual-lead (ventralis intermedius + ventralis oralis posterior) thalamic deep brain stimulation. Preoperative T_1_-weighted MRI and postoperative head CTs were used, along with applied programming settings, to estimate the volume of tissue activated for each patient. The volumes of tissue activated were then used to make voxel-wise and structural connectivity correlations with clinically observed tremor suppression. The volume of the tissue-activated analyses identified the optimal region of stimulation at the ventralis oralis posterior ventralis intermedius border intersecting with the dentato-rubro-thalamic tract. A regression model showed strong connectivity to the supplemental motor area was positively associated with tremor suppression (*r* = 0.66) in this cohort, whereas connectivity to the primary motor cortex was negatively associated with tremor suppression (*r* = −0.69), a finding opposite to that seen in ventralis intermedius deep brain stimulation for essential tremor. Comparing the structural connectivity to that of an essential tremor cohort revealed a distinct network that lies anterior to the essential tremor network. Overall, the volumes of tissue activated and connectivity observations converge to suggest that optimal suppression of multiple sclerosis tremor will likely be achieved by directing stimulation more anteriorly toward the ventralis oralis posterior and that a wide field of stimulation synergistically modulating the ventralis oralis posterior and ventralis intermedius nuclei may be more effective than traditional ventralis intermedius deep brain stimulation at suppressing the severe tremors commonly seen in complex tremor syndromes such as multiple sclerosis tremor.

## Teaser

Wong *et al*. report that supplemental motor area connectivity was positively associated with tremor suppression, whereas primary motor cortex connectivity was negatively associated with tremor suppression in unilateral dual-lead thalamic deep brain stimulation for multiple sclerosis tremor. Optimal tremor involved neuromodulation of the ventralis oralis thalamic nucleus.

## Graphical Abstract

**Graphical Abstract:**
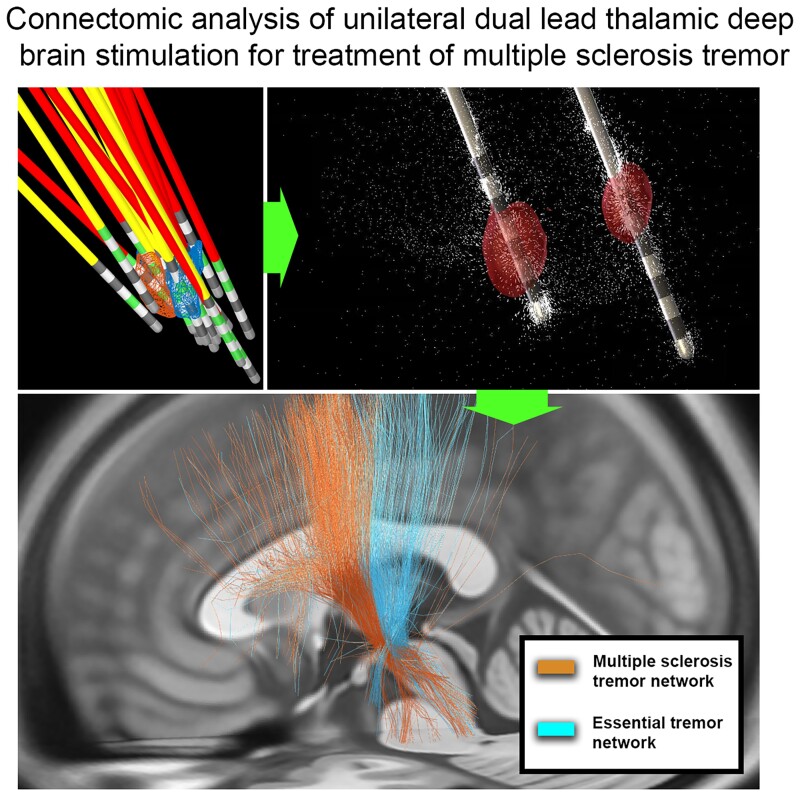

## Introduction

Tremor is a common symptom in multiple sclerosis (MS) and is estimated to have a lifetime prevalence as high as 45–75%.^1,2^ In fact, tremor was one of the three cardinal symptoms included in Charcot’s original description of the disease (‘Charcot’s triad’) along with nystagmus and staccato speech.^3^ Tremor associated with MS can have a wide range of phenomenological presentations including resting tremor, but severe postural and action tremors are more commonly the cause of significant disability. There can also be associated dystonia, ataxia and/or other debilitating neurological manifestations.^4^ The pathophysiology underpinning MS tremor has been difficult to elucidate because the causal lesions tend to be multiple and diffuse, and the disease itself is variably progressive. Consequently, defining precise MS-related anatomic correlations has been challenging.^5^ In clinical practice, tremor in MS is one of the most difficult conditions to address with pharmacologic therapy alone.^6^ Deep brain stimulation (DBS) of the ventralis intermedius (VIM) nucleus of the thalamus, the ventralis oralis posterior (VOp) nucleus of the thalamus and/or the zone incerta have all been used in an effort to address medically refractory MS tremor.^7–10^ The degree of tremor suppression post-DBS has been documented to be highly variable with high recurrence rates. Additionally, the outcomes are frequently affected by disease progression.^8^

Recently, our group published an National Institutes of Health (NIH)-funded pilot trial of unilateral dual-lead (VIM + VOp) thalamic DBS to treat medically refractory MS tremors.^11^ The goal of the present study was to apply modern connectomic techniques to data from that study in an effort to better understand treatment outcomes and optimize future DBS surgical interventions.

## Methods

Data were drawn from a previously completed single-centre, randomized, single-blind prospective MS DBS trial conducted at the University of Florida (Clinicialtrials.gov NCT00954421). The study design, surgical procedure and programming protocol have previously been described.^11^ Participants with MS and medically refractory tremor underwent dual-lead thalamic (VIM and VOp) DBS (implantation of two unilateral leads in one operative session) for tremor suppression. Participants were randomized into two groups (VIM or VOp) for single-lead stimulation and optimization for the first three months following implantation. At the 3-month visit, both leads were activated, and programming optimization continued monthly until ∼6 months. The Fahn–Tolosa–Marin Tremor Rating Scale (TRS) was collected at 3- and 6-months post-DBS implantation in the DBS OFF and DBS ON states.^12^ The 3-month TRS score was obtained during single-lead stimulation, whereas the 6-month TRS score was obtained during dual-lead stimulation. The subsequent statistical analyses in this study used the motor score of the TRS.

### Imaging analysis

The postoperative high-resolution CT head was co-registered to the preoperative MRI brain (T_1_-weighted MPRAGE) using a two-stage linear registration that was implemented in advanced normalization tools (ANTs).^13^ Preoperative and postoperative acquisitions were spatially normalized into MNI_ICBM_2009b_NLIN_ASYM template space using the symmetric normalization (SyN) registration approach as implemented in ANTs.^14^ Non-linear deformation into template space was achieved in five stages: after two linear (rigid followed by affine) steps, a non-linear (whole brain) SyN-registration stage was followed by two non-linear SyN registrations that consecutively focused on the area of interest which was defined by subcortical masks in Schoenecker *et al*.^15^ The DBS contacts on each lead were localized using the PaCER method within the lead DBS advanced imaging pipeline with manual verification and correction as needed.^16–18^ The volume of tissue activated (VTA) was estimated using finite-element modelling using the lead DBS system. An electric field was generated over a tetrahedral mesh head model that was defined as an isotropic volume with a symmetric conductivity of 0.14 S/m.^19^ An electric field threshold of 0.2 V/mm was used to define the VTA boundary.^20^

### Voxel-wise analysis

The VTAs were determined for each subject at 3- and 6-months post-DBS implantation based on the programming parameters documented at the start of the respective visit. A unique VTA was calculated for each activated lead at the two respective time points. The VTAs in the right brain hemisphere were non-linearly warped to the left hemisphere based on the MNI_ICBM_2009b_NLIN_ASYM template. Voxel-wise analysis utilizing FSL’s fslmaths function was then conducted based on previously published methods to determine the optimal stimulation location.^21,22^ Briefly, the VTAs were multiplied by the individual participant’s TRS percent improvement to create a weighted VTA heat map. A mean effect image mask was then created from the individual weighted VTA heat maps by calculating a voxel-wise mean across all subjects. We then created a ‘significant’ mean effect mask by conducting a one-sample non-parametric permutation using FSL’s threshold-free cluster enhancement function, a variance smoothing with a sigma of 2.5 mm, given small sample size and 5000 permutations.^23–25^ The significant mean effect mask was then binarized to only include voxels with an FWE-corrected *P* < 0.05. A binary cohort mask was then created to only include voxels shared by at least 40% of participants. The significant mean effect mask was multiplied with the cohort mask to generate a final significance mask, representing an improvement weighted VTA. An aggregate improvement weighted VTA that treated the VIM and VOp VTA as a single volume for each subject was also calculated using the 6-month DBS programming parameters. The centre of gravity (COG) for the improvement weighted VTAs were estimated using FSL’s COG function and reported in Montreal Neurological Institute (MNI) space. The VTAs were then visualized alongside the dentato-rubro-thalamic tract (DRTT) as defined by the DBS Tractography Atlas.^26^

### Structural connectivity analysis

The VTAs were used as seeds to calculate structural connectivity using deterministic tractography. A group averaged diffusion MRI template of 1065 healthy adults from the Human Connectome Project was utilized.^27–29^ Regions of interest (ROIs) at the cortex were defined using the Human Connectome Project Multi-modal parcellation (HCP-MMP) atlas and chosen from established structural connectivity-based segmentations of the thalamus for VIM DBS.^30,31^ Connectivity to six cortical regions were defined as follows: (i) primary visual cortex (PVC), HCP-MMP label V1; (ii) primary motor cortex (PMC), HCP-MMP label 4; (iii) Supplemental motor area (SMA), HCP-MMP label 6ma 6mp; (iv) temporal lobe (TL), HCP-MMP label PIT MT A1; (v) somatosensory complex (SSC), HCP-MMP label 1 2 3a; (vi) primary sensory cortex (PSC), HCP-MMP label 3b. Fibre tracking of each ROI was performed using 200 000 seeds and normalized to the total number of streamlines generated for each participant to define ‘connectivity’ to the ROI. Streamlines were generated via deterministic tractography in DSI Studio (http://dsi-studio.labsolver.org/).^32^ ROIs with a connectivity of 0 were discarded from further analysis. The connectivity was then analysed via linear regression to the % improvement in total TRS at 3- and 6-months post-DBS implantation. The Spearman’s rank correlation coefficient was calculated for each linear regression. Statistics were calculated using GraphPad Prism 9.1 (GraphPad Software Inc., CA, USA).

### Network comparison analysis

Discriminative fibre tract analysis was then conducted to compare the connectivity profile in this cohort with 83 essential tremor (ET) subjects treated with VIM DBS at University of Florida.^33–35^ This method utilized previously published techniques to highlight fibre tracts that are predictive of positive outcomes for each respective cohort.^33,34,36^ Through this analysis, we are able to compare the spatial differences in fibre tracts between the two groups that correlated with tremor suppression. This allowed for exploration of neuromodulation at the network level for these two tremor syndromes and elucidation of the pathological network driving MS tremor. The clinical metric for comparison was the percent TRS motor score change from baseline pre-DBS to 6-months postimplantation. Lead localization and VTAs for the ET cohort were determined using a similar method as mentioned previously. A normative connectome of 32 healthy subjects from the Human Connectome Project was designated as the structural group connectome for this analysis.^28,37^ All VTAs were binarized and independently used as a seed to estimate structural connectivity within the normative connectome. After the structural connectome was generated, the fibre tracts were refined to only include tracts that traversed at least 20% of the VTAs but no greater than 80% of the VTAs. For each fibre track generated, a two-sample *t*-test was calculated to compare the difference in percent TRS motor score change in VTAs the track traverses through versus VTAs the track does not traverse through.^34^ The corresponding *T*-score labelled each fibre as either ‘favourable’ or ‘unfavourable’ and was used to colorize each fibre track. After iterating through each fibre tract, the resulting connectome was filtered to include only tracts with a positive *T*-score. This equated to a connection with VTAs that was associated with an improvement in TRS motor score, thus representing a ‘favourable fibres’ group. The favourable fibres in the MS tremor cohort were colorized as blue, whereas the favourable fibres in the ET cohort were colorized as orange. Cortical connectivity of the two fibre groups was visualized using the Surf Ice surface rendering tool (https://www.nitrc.org/projects/surfice).

### Data availability

The data supporting the findings of this study are available from the corresponding author upon reasonable request.

## Results

Twelve participants (2 males and 10 females) were analysed from the parent study. One participant was excluded due to hardware infection ultimately requiring DBS hardware removal. Participant demographics and surgical information are summarized in Table 1. The mean ( ± SD) age at the time of DBS was 44 ( ± 14) years old. The mean ( ± SD) baseline TRS motor score was 40.4 ( ± 9.0). The mean ( ± SD) percent improvement in TRS motor score at 6 months was 29% ( ± 31%). Eight subjects carried a diagnosis of relapsing-remitting multiple sclerosis and three subjects carried a diagnosis of primary-progressive multiple sclerosis.

**Table 1:** Patient demographics and baseline clinical characteristics

Patient	Age at DBS (years)	Gender	Multiple sclerosis subtype	Initial stimulation	Surgery side	Baseline TRS motor
1	30	F	Relapsing-remitting	VIM	Left	37
2	27	M	Relapsing-remitting	VIM	Left	43
3	49	F	Relapsing-remitting	VOp	Left	30
4	54	F	Primary-progressive	VOp	Right	34
5	47	F	Relapsing-remitting	VIM	Left	49
6	51	F	Relapsing-remitting	VOp	Left	41
7	36	F	Relapsing-remitting	VIM	Left	50
8	58	F	Relapsing-remitting	VIM	Left	26
9	23	F	Primary-progressive	VIM	Left	34
10	40	F	Relapsing-remitting	VOp	Right	42
11	72	F	Primary-progressive	VOp	Left	58

**Figure 1:**
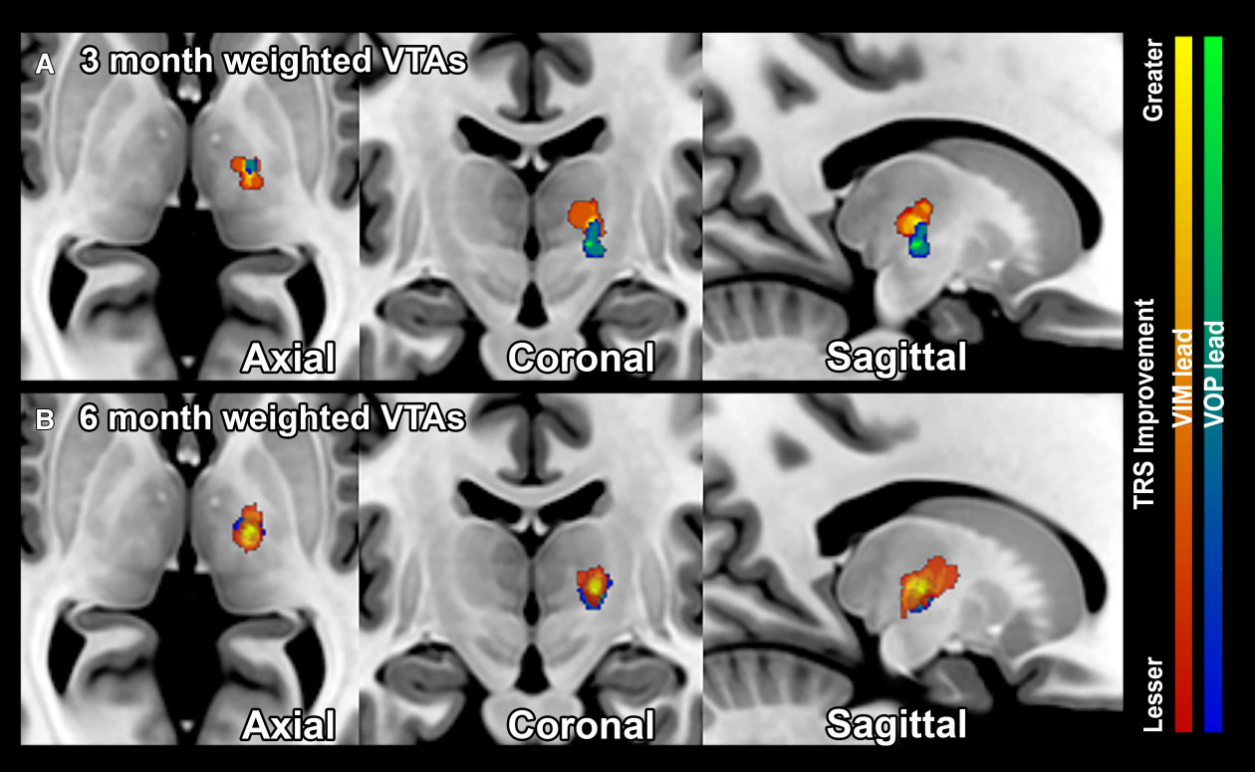
**Improvement weighted VIM and VOp VTAs in dual-lead DBS.** The improvement weighted VTAs are shown as an improvement heat map gradient for the VIM lead (red-yellow) and VOp (blue-green) at (**A**) 3-months (single-lead stimulation) and (**B**) 6-months (dual-lead stimulation) post-DBS implantation. The VTAs are superimposed upon a normalized MRI T1 sequence (MNI152 NLIN 2009).

Improvement weighted VTAs of VIM and VOp stimulation at 3- and 6-months post-DBS implantation are summarized in Fig. 1. The COG for the 3-month VIM and VOp VTAs are (−14.0, −16, 4.5) and (−14.5, −17, −3), respectively. The COG for the 6-month VIM and VOp VTAs is (−16, −11.5, 4) and (−15, −15, −1.5), respectively. The COG for the aggregate 6-month VTAs is (−16, −12, 2.5).

**Figure 2:**
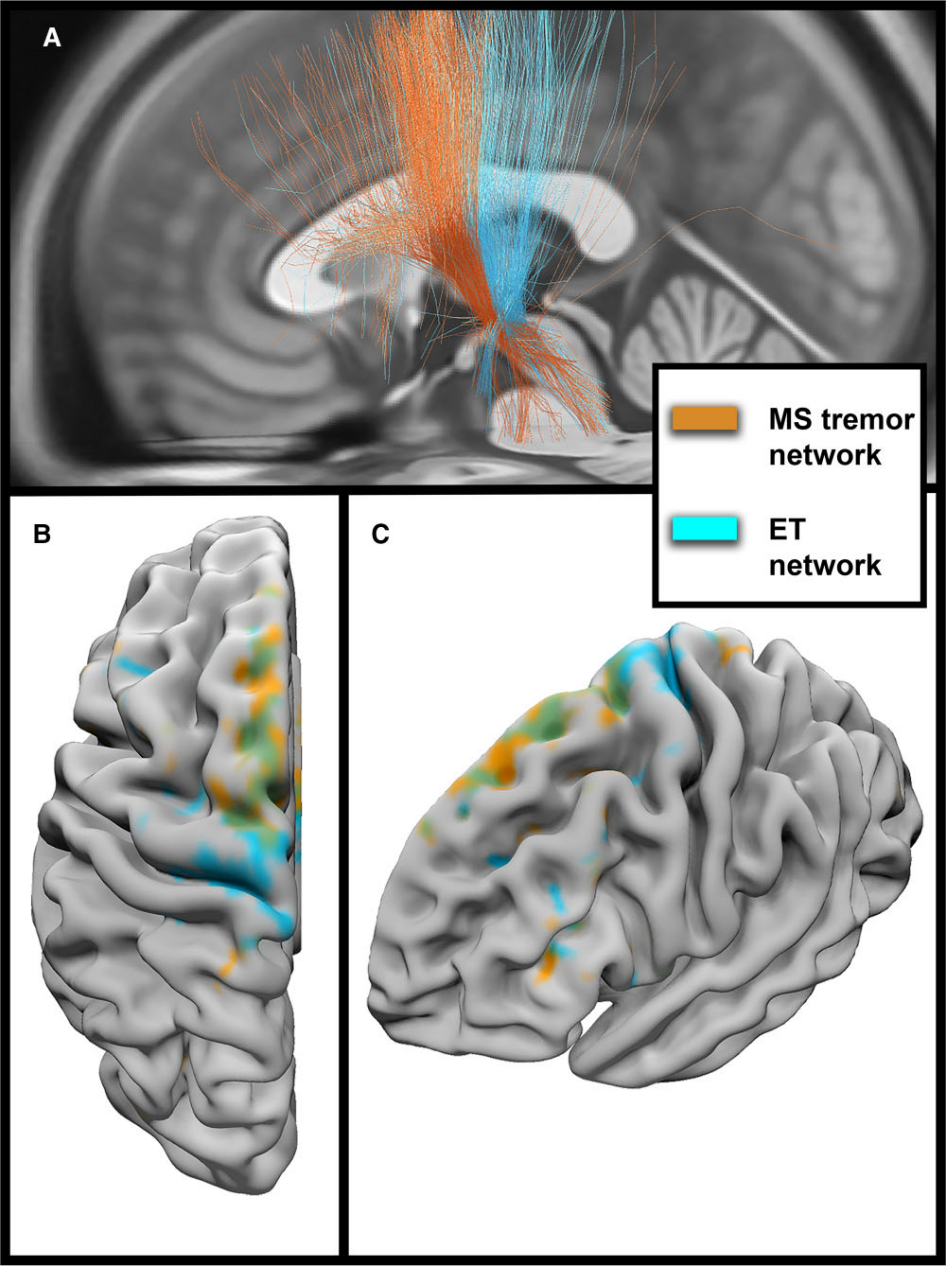
**Network differences in DBS for MS tremor and ET.** Identification of favourable fibres for the MS tremor cohort (orange) and the ET cohort (blue) are shown in (**A**). Modulation of these fibres is associated with greater tremor suppression. Cortical connectivity of the two tracts is rendered over an ICBM152 left hemisphere model and shown from an axial view in (**B**) and an oblique view in (**C**). The green regions represent overlapping areas of connectivity between the two groups.

A network level comparison of ‘favourable fibres’ between the MS cohort and ET cohort can be seen in Fig. 2A. The fibres associated with greater tremor suppression in the MS cohort lie anterior to the favourable fibres of the ET cohort. Cortical connectivity differences of the favourable fibres can be seen in Fig. 2B and C. Although there are overlapping regions of modulation, tremor suppression in the MS cohort is more associated with premotor cortex modulation, whereas tremor suppression in the ET cohort is associated with PMC modulation.

**Figure 3:**
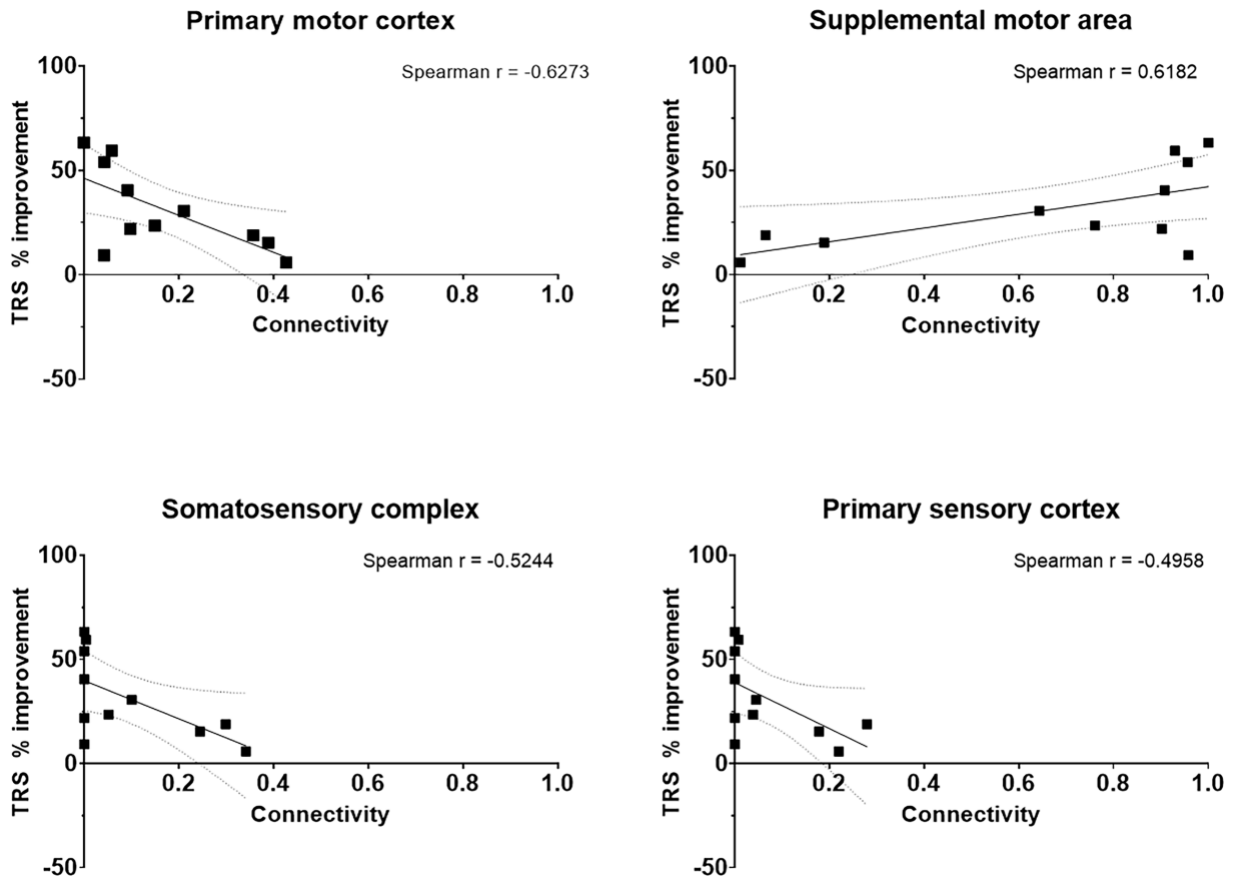
**Connectivity profile of dual-lead thalamic DBS at 3 months.** The connectivity profile of the VIM and VOp leads are shown as a linear regression with respect to percent improvement in TRS motor score at 3 months postimplantation. The dotted lines represent the 95% confidence band of the best fit line.

**Figure 4:**
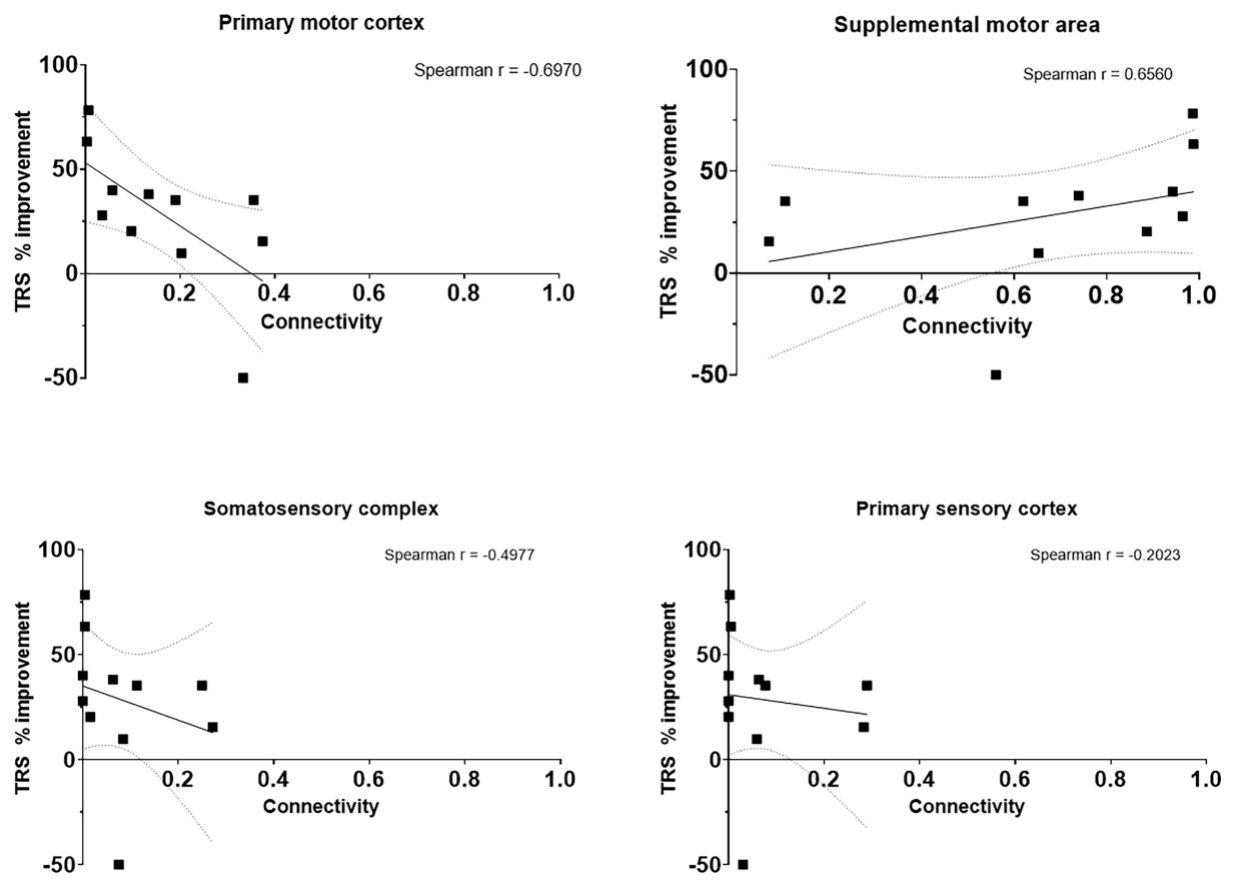
**Connectivity profile of dual-lead thalamic DBS at 6 months.** The connectivity profile of the VIM and VOp leads are shown as a linear regression with respect to percent improvement in TRS motor score at 6 months postimplantation. The dotted lines represent the 95% confidence band of the best fit line.

Cortical connectivity relationships at 3- and 6-months post-DBS implantation can be observed in Figs 3 and 4 respectively. There was no connectivity to the PVC or TL in all 11 participants. There was a positive correlation between SMA connectivity and tremor suppression, whereas a negative correlation was observed with PMC, SSC and PSC connectivity.

**Figure 5:**
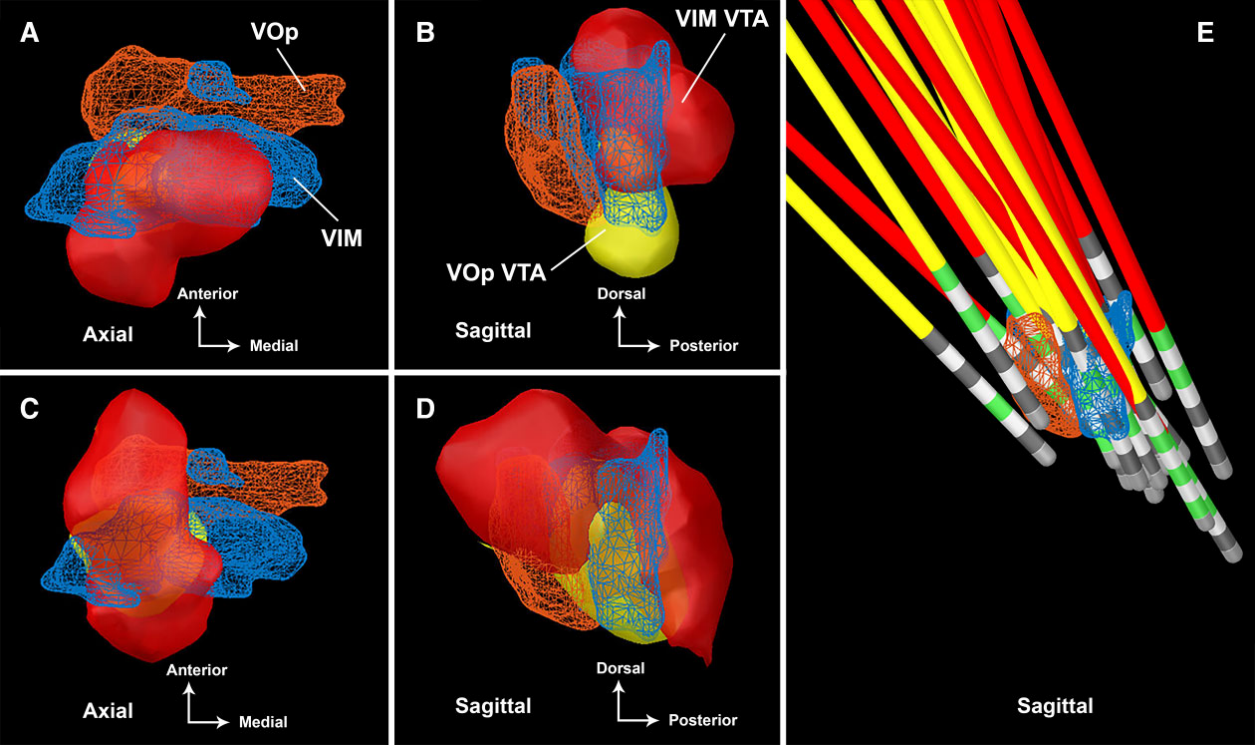
**Spatial relationship of dual-lead DBS VTAs.** The binarized aggregate VTAs for the VIM-targeted (red) and VOp-targeted (yellow) DBS leads are shown with respect to the VIM nucleus (blue) and VOp nucleus (orange) at (**A** and **B**) 3 months and (**C** and **D**) 6 months post-DBS implantation. Localization of the VIM leads (red) and VOp leads (yellow) are shown in (**E**) from the sagittal plane. The active contacts for each lead are denoted in green. The thalamic nuclei are defined from the DISTAL.^38^

**Figure 6:**
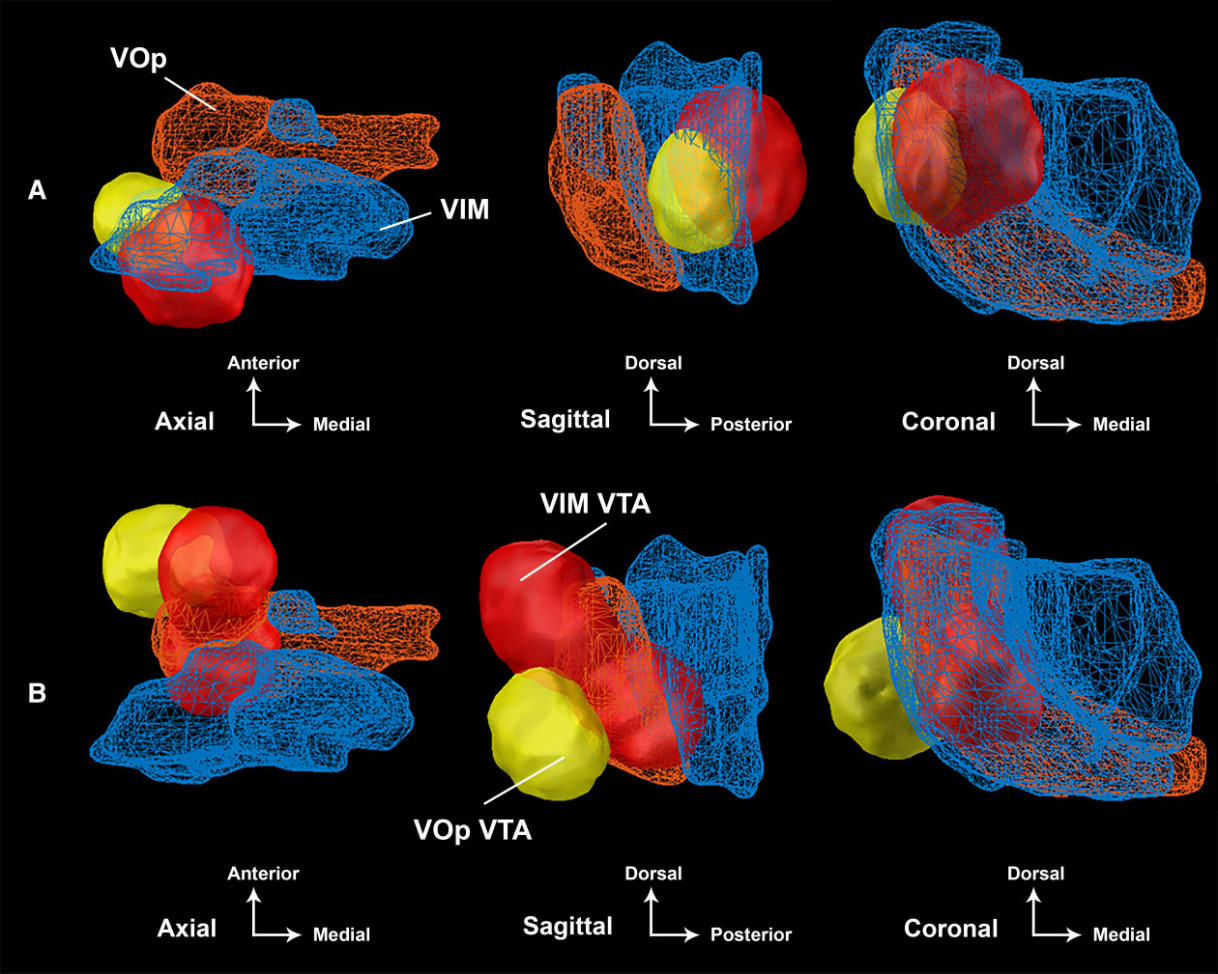
**A differential response to dual-lead DBS for MS tremor.** The spatial differences for the VIM-targeted (red) and VOp-targeted (yellow) VTAs are shown for the (**A**) non-responder subject and (**B**) super-responder subject at 6 months postimplantation. The VIM nucleus (blue) and VOp nucleus (orange) are defined from the DISTAL.^38^

Binarization of the improvement weighted VTAs were superimposed on the VIM and VOp as defined by the DBS Intrinsic Atlas (DISTAL), and these have been displayed in Fig. 5.^38^ One participant (Subject 8) experienced worsening of tremor at 6-months post-DBS implantation (−50% worsening) and was labelled a non-responder. Her connectivity profile demonstrated strong connectivity to the PMC (0.33) and weaker connectivity to the SMA (0.56). The non-responder VTAs have been summarized in Fig. 6A. Another participant (Subject 1) experienced significant improvement of tremor at 6-months post-DBS (78% improvement) and was labelled a super-responder. Her connectivity profile demonstrated minimal connectivity to the PMC (0.03) and very strong connectivity to the SMA (0.96). The super-responder VTAs are shown in Fig. 6B.

**Figure 7:**
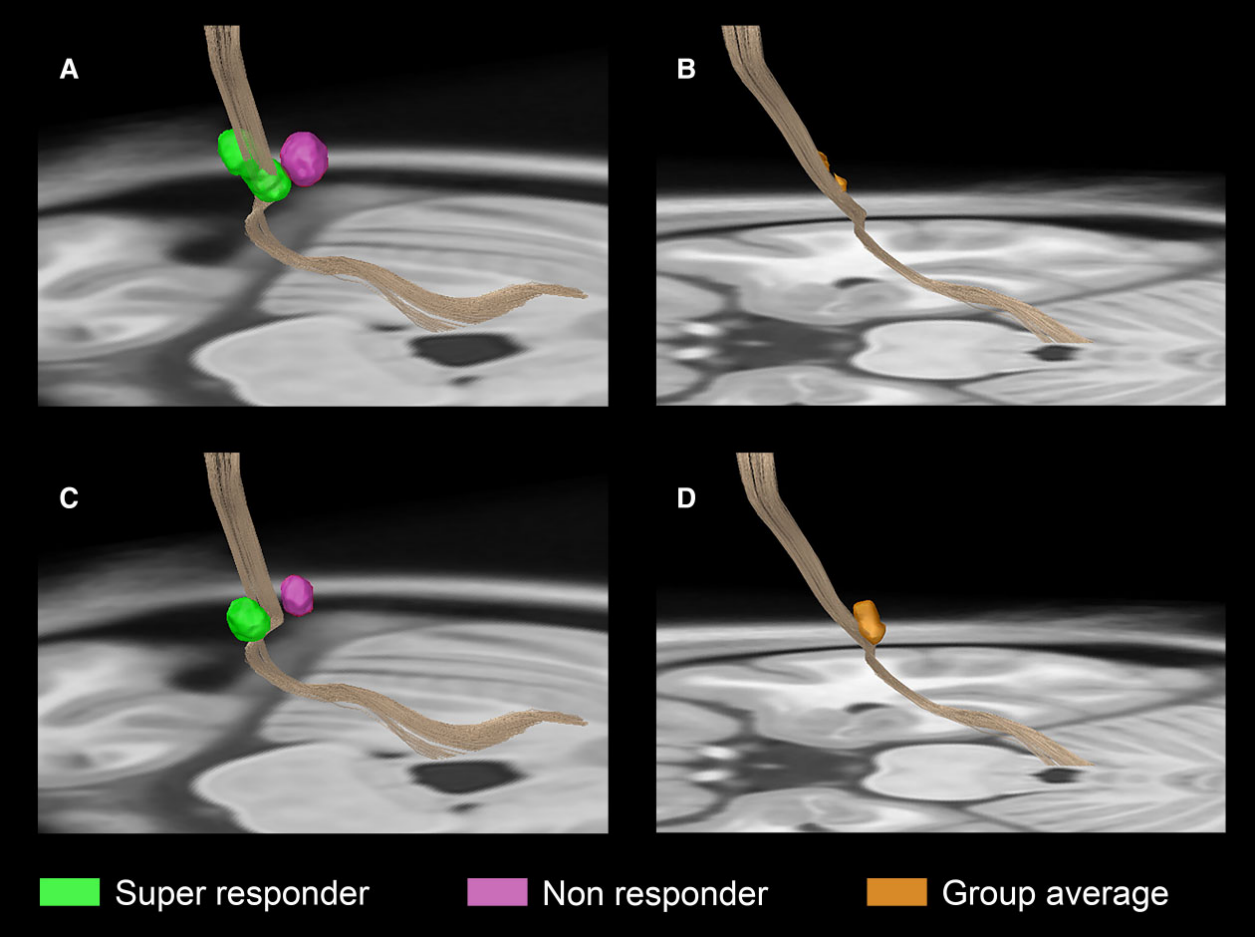
**Connectivity to the DRTT in dual-lead thalamic DBS.** The VIM-targeted and VOp-targeted VTA at 6 months postimplantation are shown in (**A**) and (**C**) for the super-responder subject (green) and non-responder subject (magenta), respectively. The group averaged VIM-targeted and VOp-targeted VTA excluding the non-responder is shown in (**B**) and (**D**). The fibre tract depicted in brown is the decussating DRTT as defined by the DBS Tractography Atlas.^26^

The spatial relationship of the super-responder and non-responder VTAs for the VIM and VOp leads located within the (DRTT can be seen in Fig. 7A and C, respectively.^26^ The improvement weighted VTAs for the VIM and VOp leads are represented in Fig. 7B and D, respectively.

## Discussion

This study applied modern connectomic analyses to the cohort of MS tremor subjects who participated in the parent NIH trial. The multifocality and broad distribution of MS lesions add to the complexity of targeting. The optimal target for DBS has been elusive with many studies yielding mixed results.^39^ With the single-lead stimulation, we observed that the VIM and VOp VTAs modulated a similar ‘column’ of thalamic tissue as noted by the congruent COGs, primarily differing in the *z*-axis. After dual-lead stimulation was introduced, the VTAs shifted anteriorly and dorsally into the VOp nucleus as seen in Fig. 5. This anterior location is in front of the VIM nucleus, which is the traditional DBS tremor target. In the anterior–posterior plane, this spatial difference was highlighted by the position of the non-responder VTA in the centre of the VIM nucleus, whereas the super-responder VTA was located at the anterior aspect of the VOp nucleus (Fig. 6). These findings suggest that with the single-lead stimulation, tremor suppression is primarily driven by VIM stimulation. However, with dual-lead stimulation, the expanded volume evoked a drive to shift the electric field anteriorly into the VOp nucleus.

At the network level, strong cortical connectivity to the SMA was associated with improvement in MS tremor. This finding was similar to previous tractography studies conducted in a population of ET subjects that demonstrated a correlation between SMA connectivity and tremor suppression.^31^ Our data importantly revealed a negative correlation with PMC connectivity and tremor suppression, a finding opposite to that observed in the ET cohort. This point is emphasized in Fig. 2A as identification of ‘favourable fibres’ for neuromodulation in the MS cohort illustrates a distinct set of fibre tracts when we compare them to the ET cohort. Fig. 2B and C also highlight the importance of connectivity to the PMC in ET DBS as observed in previous studies, but no such association was found in the MS tremor cohort.^31^ These findings suggest that the expanded VTA provided by unilateral dual-lead DBS may actually be necessary at least in some cases to achieve the required magnitude of thalamic modulation for tremor suppression. Further studies will be needed to confirm this hypothesis.

In MS tremor, historically VIM was targeted under the premise that tremor generation was likely related to dysfunction in the cerebello-thalamo-cortical circuitry.^8,40^ The lack of robust response when compared with primary tremor disorders has opened the door for alternative pathophysiological hypotheses.^41^ Investigations of the VOp as an alternative target were previously based on the idea that it was a pallidal receiving area and that it may be possible to spread activating current posteriorly into the cerebellar VIM receiving area either with an intentionally placed single lead or with the use of unilaterally placed dual leads.^42^ To date, there has been insufficient evidence comparing VIM vs. VOp DBS for MS tremor. A recent meta-analysis concluded that DBS was safe and effective; however, due to the heterogeneity of the data and small sample sizes, target-specific comparisons were not possible.^39^ By utilizing connectomic analyses, we were able to observe that the best programming configuration in VIM DBS for MS tremor may actually be activating the VOp nucleus area and its connections. This finding may also partially explain the past difficulties of comparing VIM vs. VOp DBS for MS tremor and disentangling the two areas.

Analysis of the fibre tracts with proximity to the DRTT was associated with greater tremor suppression as displayed in Fig. 7. The DRTT travels from the dentate nucleus in the cerebellum, across the midbrain, passing by the contralateral red nucleus and through the VIM and VOp before projecting onto the PMC.^26^ Radiographically, it can be partitioned into decussating dentato-rubro-thalamic tract (dDRTT) and non-decussating dentato-rubro-thalamic tract (ndDRTT) branches with the dDRTT generally lying more anterior within the ventral thalamus (VIM and VOp), whereas the ndDRTT is more posterior and medial (closely associated with the VIM).^26^ The DRTT depicted in Fig. 7 is representative of the decussating branch. Structurally, the anterodorsal preference and proximity to the dDRTT may also facilitate neuromodulation of pallidothalamic tracts and another fibre bundle known to project to the VOp nucleus. Tsuboi *et al*.^43^ showed that modulation of the pallidothalamic tract was significantly associated with improvement in dystonic tremor but not in ET. Given the phenomenological complexity of MS tremor and the disseminated nature of lesion burden in MS, it is conceivable that multiple pathologic networks contribute to the overall tremor syndrome. The findings from our spatial and connectomic analyses may suggest that the pathology underpinning MS tremor may share similarities with dystonic tremor at the network level. The findings collectively suggest that although modulation of the cerebello-thalamo-cortical circuitry was associated with tremor suppression, there exists a distinct connectivity profile difference in optimal thalamic stimulation in MS tremor when compared with ET. Triangulation of the concordance between the VTA and connectivity observations highlights that this difference likely lies within the VOp nucleus. This hypothesis may explain the lack of robust outcomes in previous attempts to treat MS tremor with a single VIM lead.

This study had several limitations. First, it was a *post hoc* analysis of a previously published NIH prospective clinical trial and thus was not designed or powered to detect connectivity differences between surgical targets. Additionally, the sample size for this study was small, and the follow-up period was limited to 6 months. We could not control for MS progression over the study period. Several technical assumptions were made as part of the imaging analysis. The brain was modelled as an isotropic medium with uniform conductivity used for the VTA modelling. Structural data were normalized into MNI template space and analysed at the group level with MNI-based atlases. The study also utilized normative connectome data as opposed to patient-specific data. Although the debate of normative versus patient-specific data is ongoing, many studies have revealed that normative data yields similar results to patient-specific data.^44^ We cannot, however, at this time draw this conclusion based on the data from this small cohort. Despite these limitations, important connectomic data were derived from our investigation.

In conclusion, the DRTT is a well-studied structure thought to be highly involved with tremor genesis. However, in complex tremor disorders such as MS tremor, there may be additional pathological circuitry involved and possibly multiple tremor oscillators. In the present study, we show that unilateral dual-lead thalamic DBS provided a greater volume of stimulation and that there was a negative correlation between PMC connectivity and tremor suppression in this MS tremor cohort, a finding opposite to that observed in the ET cohort. Our findings here suggest that more anterior thalamic stimulation (VOp), incorporating both the DRTT and pallidothalamic circuits (with connectivity to the supplementary motor area), may be important for improving the outcomes of DBS for MS tremor.

## Abbreviations

ANTs =: advanced normalization tools
COG =: centre of gravity
DBS =: deep brain stimulation
DISTAL =: DBS Intrinsic Atlas
DRTT =: dentato-rubro-thalamic tract
ET =: essential tremor
FSL =: FMRIB Software Library
TRS =: Fahn–Tolosa–Marin Tremor Rating Scale
HCP-MMP =: Human Connectome Project Multi-modal parcellation
MNI =: Montreal Neurological Institute
MPRAGE =: magnetization prepared rapid gradient echo
MS =: multiple sclerosis
PaCER =: Precise and Convenient Electrode Reconstruction for Deep Brain Stimulation
ROI =: region of interest
SyN =: symmetric normalization
VIM =: ventralis intermedius
VOp =: ventralis oralis posterior
VTA =: volume of tissue activated
